# Costs of relaparotomy on-demand versus planned relaparotomy in patients with severe peritonitis: an economic evaluation within a randomized controlled trial

**DOI:** 10.1186/cc9032

**Published:** 2010-05-27

**Authors:** Brent C Opmeer, Kimberly R Boer, Oddeke van Ruler, Johannes B Reitsma, Hein G Gooszen, Peter W de Graaf, Bas Lamme, Michael F Gerhards, E Philip Steller, Cecilia M Mahler, Huug Obertop, Dirk J Gouma, Patrick MM Bossuyt, Corianne AJM de Borgie, Marja A Boermeester

**Affiliations:** 1 Department of Clinical Epidemiology, Biostatistics and Bioinformatics, Academic Medical Center, Meibergdreef 9, 1105 AZ, Amsterdam, The Netherlands; 2 Department of Surgery, Academic Medical Center, Meibergdreef 9, 1105 AZ, Amsterdam, The Netherlands; 3 Department of Surgery, Universitary Medical Center Utrecht, Heidelberglaan 100, 3584 CX, Utrecht, The Netherlands; 4 Department of Surgery, Reinier de Graaf Gasthuis, Reinier de Graafweg 3-11, 2625 AD, Delft, The Netherlands; 5 Department of Surgery, Gelre Hospital, Albert Schweitzerlaan 31, 7334 DZ, Apeldoorn, The Netherlands; 6 Department of Surgery, Onze Lieve Vrouwe Gasthuis, Oosterpark 9, 1091 AC, Amsterdam, The Netherlands; 7 Department of Surgery, Sint Lucas Andreas Hospital, Jan Tooropstraat 164, 1061 AE, Amsterdam, The Netherlands

## Abstract

**Introduction:**

Results of the first randomized trial comparing on-demand versus planned-relaparotomy strategy in patients with severe peritonitis (RELAP trial) indicated no clear differences in primary outcomes. We now report the full economic evaluation for this trial, including detailed methods, nonmedical costs, further differentiated cost calculations, and robustness of different assumptions in sensitivity analyses.

**Methods:**

An economic evaluation was conducted from a societal perspective alongside a randomized controlled trial in 229 patients with severe secondary peritonitis and an acute physiology and chronic health evaluation (APACHE)-II score ≥11 from two academic and five regional teaching hospitals in the Netherlands. After the index laparotomy, patients were randomly allocated to an on-demand or a planned-relaparotomy strategy. Primary resource-utilization data were used to estimate mean total costs per patient during the index admission and after discharge until 1 year after the index operation. Overall differences in costs between the on-demand relaparotomy strategy and the planned strategy, as well as relative differences across several clinical subgroups, were evaluated.

**Results:**

Costs were substantially lower in the on-demand group (mean, €65,768 versus €83,450 per patient in the planned group; mean absolute difference, €17,682; 95% CI, €5,062 to €29,004). Relative differences in mean total costs per patient (approximately 21%) were robust to various alternative assumptions. Planned relaparotomy consistently generated more costs across the whole range of different courses of disease (quick recovery and few resources used on one end of the spectrum; slow recovery and many resources used on the other end). This difference in costs between the two surgical strategies also did not vary significantly across several clinical subgroups.

**Conclusions:**

The reduction in societal costs renders the on-demand strategy a more-efficient relaparotomy strategy in patients with severe peritonitis. These differences were found across the full range of healthcare resources as well as across patients with different courses of disease.

**Trial Registration:**

ISRCTN51729393

## Introduction

Secondary peritonitis or abdominal sepsis is a serious condition with high in-hospital mortality (estimates vary between 20% and 60%) and considerable major disease-related morbidity [1-4]. Patients with severe peritonitis require intensive monitoring and medical treatment, often including lengthy ICU stays. With an estimated incidence for the United States of 9.3 cases of patients with secondary peritonitis per 1,000 emergency hospital admissions [5], these patients incur substantial costs to the healthcare system.

The initial treatment of abdominal sepsis consists of an emergency laparotomy aimed at eliminating the source of the infection. Thereafter, two surgical strategies are used world-wide: planned relaparotomy or relaparotomy on demand. In the planned strategy, a relaparotomy is performed every other day (24 to 36 h) until findings are negative for (ongoing) peritonitis. This strategy may incur the risk of potential surgery-related complications. The on-demand strategy uses 'watchful waiting,' in which a relaparotomy is performed only in those patients showing clinical deterioration or lack of improvement. Fewer relaparotomies are likely to be performed with this strategy [3], which may benefit the already critically ill patients, but may lead to a potentially harmful delay.

The debate about the preferred relaparotomy strategy (on-demand versus planned) in these patients is longstanding, with both strategies having their proponents. We recently published the results of the first randomized trial comparing these two surgical strategies and demonstrated that patients in the on-demand group did not have a significantly lower rate of adverse clinical outcomes compared with the planned group [6]. However, the economic evaluation from a healthcare perspective showed that total costs after 12 months of follow-up were estimated at 23% lower per patient in the on-demand group (€62,741 (US, $86,077)) as compared with a planned-relaparotomy strategy (€81,532 (US, $111,858)).

Here we present the economic evaluation comparing costs generated by an on-demand and a planned-relaparotomy strategy from a societal perspective. More details are reported, regarding both methods and the clinical process driving these costs. Sensitivity analyses were performed to evaluate the robustness of the findings for several assumptions and methodologic choices. Furthermore, differences in costs are assessed across patients with different clinical characteristics and courses of disease.

## Materials and methods

### Design and eligibility

This economic evaluation was part of the RELAP trial, a randomized controlled multicenter trial comparing an on-demand relaparotomy strategy with a planned-relaparotomy strategy in patients with severe peritonitis. Details about the design, conduct, and major clinical findings of this trial were reported elsewhere [6]. In brief, we included patients diagnosed with secondary peritonitis and requiring an emergency laparotomy, an Acute Physiologic and Chronic Health Evaluation (APACHE) II score greater than 10 in the initial 24-h period [7], and aged between 18 and 80 years [6]. The clinical diagnosis of peritonitis was confirmed during the index laparotomy. Excluded were patients with continuous ambulatory peritoneal dialysis (CAPD)-related peritonitis and pancreatitis. Specialized randomization software was used to allocate patients centrally, with stratification by study site and APACHE-II classification as a minimization factor [8]. The operating surgeon was unaware of the allocated treatment strategy while performing the initial emergency laparotomy. The study was approved by the medical ethics committees of all participating centers.

The appropriate type of economic evaluation is conditional on the results of the primary end points (mortality, major disease-related morbidity) and health-related quality-of-life (HR-QoL). In case of one clearly superior strategy, a cost-effectiveness analysis (CEA) would be required to combine clinical and economic outcomes. In case of comparable outcomes in clinical effectiveness, a cost-minimization analysis (CMA) would suffice [9]. As clinical end points in this study were comparable or even in favor of the on-demand strategy, this economic evaluation was set up as a cost-minimization analysis.

### Economic evaluation

Healthcare utilization and other resources were prospectively documented for individual patients [10], by using registration forms of the clinical study, and by acquiring data from additional sources where needed. The horizon for the economic evaluation was 12 months after the initial emergency laparotomy.

The cost analysis was set up from a societal perspective, which consists of three cost categories [11]. These include direct medical costs, direct nonmedical costs, and indirect costs [9,12]. Direct medical costs are generated by healthcare utilization and include hospital and ICU admission periods, therapeutic and diagnostic procedures, medication, and visits to primary and paramedical healthcare providers after discharge. Direct nonmedical costs are generated by expenses for travel to and from healthcare providers. Indirect costs are associated with loss of productivity due to impaired ability to work [12]. This societal perspective allows a more complete economic evaluation as compared with a healthcare perspective, used for earlier cost estimates [6].

To exclude potential protocol-driven costs, we assessed the extent to which procedures and follow-up visits are part of the usual care for these patients or are relevant only for the conduct of the trial. Basically, some surgical and diagnostic interventions formed protocols for the study, but this was because they are inherent to the surgical strategy; other procedures and interventions were at the discretion of the surgeon, reflecting usual care.

### Resource utilization

Data on resource utilization during the index hospital admission included the number of surgical interventions (including relaparotomies), percutaneous drainage procedures, diagnostic procedures (abdominal computer tomography (CT), ultrasound (US), plain radiograph of the abdomen), postoperative hospital stay from index laparotomy onward, and ICU stay. The initial summary cost analyses reported with the main clinical findings [6] were further refined by differentiating between relaparotomies (performing further abdominal repair) and second-look relaparotomies; the latter were assumed to generate fewer costs.

All data were registered alongside the clinical study. Resource utilization after discharge was documented in specifically developed, self-administered questionnaires that were sent to surviving discharged patients at 3, 6, 9, and 12 months of follow-up. Patients reported use of primary, secondary, and paramedic healthcare services. Employment status and absence from paid work was also documented by using the Health and Labour Questionnaire [13]. Nonresponse to the mailed questionnaires was followed by a reminder by phone, and a new questionnaire after 1 month.

### Unit costs

Estimates of unit costs were derived from different sources [see Additional File 1]: Dutch reference data from the handbook of the Dutch Health Council [12,14]; Dutch pharmaceutical unit cost listings [15]; insurance reimbursement fees [16]; top-down cost calculations; and bottom-up cost calculations performed by the authors. All costs were set at the year 2004 price level by using the price index rate for the Dutch healthcare sector.

### Costs: calculations

Costs were calculated for individual patients by multiplying actually used healthcare resources and unit costs. Data concerning postdischarge healthcare utilization were not always complete because of partial or nonresponse to the self-administered questionnaires. In these cases, the average resource utilization for an out-of-hospital day was estimated within each study/treatment group, and extrapolated over the total out-of-hospital period. Productivity costs due to illness or recovery in patients younger than 65 years were estimated based on patient-reported absences from paid work. Productivity costs were calculated by using a friction cost approach, which assumes that after a friction period (154 days), each employee is replaced in the workforce [12].

### Statistical analyses

All analyses were performed according to the intention-to-treat principle [17]. Data management and analyses were performed with SPSS 12 (SPSS Inc., Chicago, IL), MS Excel 2003, and SAS 9.1 (SAS Institute, Cary, NC).

Mean volumes of resource utilization and associated costs during the index admission and 1-year follow-up were estimated for the two surgical strategies. Differences in volumes were tested for significance by using a nonparametric Wilcoxon Mann-Whitney test.

Total costs per patient were estimated as the sum of direct medical costs, direct nonmedical costs, and indirect costs. Differences in total costs between the two surgical strategies were tested based on their geometric means [18].

Robustness of our results were evaluated in sensitivity analyses regarding the extent to which cost differences can be attributed to costs of relaparotomies alone, as well as for unit costs for a range of cost drivers [5,6]. We evaluated whether differences in costs were consistent across patients with varying clinical conditions, or whether these differences were more pronounced in more severely ill patients. We hypothesized that the total costs are a proxy for severity of the clinical condition, as more severely ill patients would require more-complicated care and more-intensive treatment, thereby generating more resource utilization and costs. Based on this assumption, we compared the distribution of costs across patients with different clinical conditions (from those patients with a relatively quick recovery and less resource utilization to patients with a more-severe course of disease with slower recovery and extensive use of healthcare resources) between the two strategies. The comparison was graphically presented by ranking patients within each study group by their total costs, and comparing total costs of patients with similar ranks.

Finally, we hypothesized that the relative difference between the on-demand relaparotomy strategy and the planned strategy could be different across clinical subgroups: between patients with and without any major comorbidity (defined as malignancy, cardiovascular disease, respiratory disease, renal disease, or diabetes), patients with high (>20) versus patients with lower (11-20) APACHE-II scores, and between patients surviving and those who died before 12 months of follow-up. A linear regression model was used, with the log-transformed costs as the outcome measure to improve the normality of the residuals required in such models. Differences between clinical subgroups were estimated and statistically tested by adding this as an interaction effect with the type of surgical strategy to the model.

## Results

### Main clinical findings

In total, 229 patients were correctly randomized and included in this study. In both surgical strategies, one patient withdrew informed consent, and one patient was lost to follow-up, meaning that data on the initial admission were available for 229 patients (114 on-demand and 115 planned strategy), and data on the 1-year follow-up, for 225 patients (112 on-demand and 113 planned strategy).

Demographic and clinical baseline characteristics of these randomized patients are presented in Table 1, together with a summary of the main clinical outcomes. The results show that morbidity and mortality were comparable. More details on the clinical outcomes can be found in the trial publication of clinical outcomes [6].

**Table 1:** Patient characteristics and summary of main clinical outcomes in the on-demand and planned-relaparotomy groups [6]

Characteristic	On demand	Planned
APACHE II >20, *n *(%)^a^	16 (14)	19 (17)
Mannheim Peritonitis Index [28], mean (95% CI)^a^	27 (23 to 32)	29 (24 to 33)
One or more comorbidity present, *n *(%)^a^	64 (56)	72 (63)
		
Mortality at 1 year, *n *(%)^b^	32 (29)	41 (36)
Major morbidity in survivors, *n *(%)^c^	32 (40)	32 (44)

^a^At index admission (*n* = 229); ^b^at follow up (*n* = 225); ^c^in surviving patients (*n* = 152).

### Resource utilization and costs

Data available for analysis from the index admission and follow-up admissions were available for all patients. Additional data on use of healthcare resources outside the hospital (outpatient care and other healthcare providers), travel, and absence from work were reported for at least one follow-up period by 76 patients in the on-demand group and 74 patients in the planned group. For both strategies, mean costs per out-of-hospital day were estimated and extrapolated to patients who did not report this part of the follow-up.

Results of the cost analyses are presented by reporting mean volumes, total costs, mean costs per patient per strategy for the on-demand group and the planned group for resource utilization during the index admission, and follow-up (Table 2). Mean costs per patient associated with relaparotomy procedures during the index admission were estimated as €4,617 (index laparotomy plus 113 relaparotomies in 114 patients) for the on-demand group and €6,641 (index laparotomy plus 233 relaparotomies in 115 patients) for the planned group (*P *< 0.001). Although in the on-demand group, significantly fewer relaparotomies comprised additional surgical procedures (42 in the on-demand and 54 in the planned-relaparotomy group; *P *= 0.022), the associated mean costs per patient (€1,211 for the on-demand group and €1,543 for the planned group) of these additional procedure differed only marginally (€332).

**Table 2:** Mean use of resources and costs in the on-demand and planned-relaparotomy groups during index admission and follow-up until 1 year after randomization

		OD (*n* = 114)		PR (*n* = 115)		Difference (PR-OD)
	Unit	Mean volume	Mean costs p.p. (€)	Mean volume	Mean costs p.p.(€)	Mean costs p.p. (€)
**Direct medical costs**						
						
*Index admission*						
Admission						
Ward stay, index (excl ICU)	Day	26	11,609	27	11,784	175
ICU stay	Day	12	21,040	18	31,248	10,208
						
Interventions						
(re)Laparotomy						
Index laparotomy	Procedure	1.0	2,267	1.0	2,267	0
Second-look	Procedure	0.62	1,139	1.5	2,831	1,692
with other surgical procedures	Procedure	0.37	1,211	0.47	1,543	332
Percutaneous drainage	Procedure	0.41	123	0.67	199	84
						
Diagnostic CT and cultures						
CT	Procedure	1.2	302	1.4	341	39
Microbiology	Cultures	43	586	58	792	206
						
Medication and other materials						
Antibiotic therapy (excl ICU)	Day	6.0	474	6.1	619	145
Enterostomy care^b^	Day	24	741	29	917	176
Blood products	Unit	0.61	89	1.04	178	89
Mechanical ventilation	Day	8.3	3,080	12	4,360	1,280
						
SUBTOTAL			*42,661*		*57,079*	*14,418*
						
Follow-up						
		OD (*n* = 112)		PR (*n* = 113)		
Inpatient care						
Ward stay, follow up	Day	9.5	4,280	11.8	5,083	803
Elective surgery	Procedure	0.39	875	0.51	1,163	288
Percutaneous drainage	Procedure	0.08	24	0.11	32	84
						
Outpatient care						
Specialist consultation^a^	Visit	16.3	1,297	16.7	1,333	36
CT abdomen	Procedure	0.07	18	0.03	7	-11
US abdomen	Procedure	0.05	5	0.03	2	-2
Plain radiograph	Procedure	0.18	8	0.12	5	-3
Enterostomy care^b^	Day	140	4,449	150	4,767	318
						
Other health care providers						
Primary care physician^a^	Visit	9.4	194	8.5	175	-19
Company doctor^a^	Visit	1.8	42	1.4	31	-10
Paramedical specialist^a^	Visit	29	675	29	667	-7
District nurse^a^	Hour	45	1,836	71	2,947	1,111
Rehabilitation center^a^	Day	19	6,480	23	8,040	1,560
						
SUBTOTAL			*20,183*		*24,252*	*4,069*
						
Direct nonmedical costs						
Travel costs^a^	Km	395	71	393	71	0
						
Indirect costs						
Absence from paid work^a^	Day	70	2,854	50	2,048	-806
						
Total costs (€)			**65,768**		**83,450**	**17,682**

^a^PR *n* = 74/OD *n* = 76: number of patients on whom analyses are based. Average costs per patients per day outside the hospital within each arm were extrapolated to patients for whom this information was not available. ^b^PR *n* = 53 patients with enterostomy; OD *n* = 49 patients with enterostomy; volume, average amount of resources used per patient; total costs, total costs of resource use of all patients; mean costs, average cost per patient; difference, difference between PR and OD (positive values in favor of OD).

Higher costs generated by additional US- or CT-guided percutaneous drainages (€147 (27% received PCD) for the on-demand group and €233 (39% received PCD; *P *= 0.038)) for the planned group) did not compensate for the lower numbers of relaparotomies in the on-demand group.

As a large majority of patients were admitted to the ICU, substantial costs were generated by the ICU stay. Mean estimated costs per patient generated by the ICU stay were €21,040 (90% of the patients; mean stay, 12 days) in the on-demand group and €31,248 (94% of the patients; mean stay, 18 days; *P *= 0.001) for the planned-relaparotomy group (mean difference, €10,208). Costs associated with mechanical ventilation showed a similar picture, with mean costs estimated as €3,080 (mean, 8.3 days) for the on-demand group and €4,360 (mean, 12 days; *P *= 0.004) for the planned group (mean cost difference, €1,280). Costs generated by hospital stay on the ward (excluding ICU days) were estimated as €11,609 for the on-demand group and €11,784 for the planned-relaparotomy group. Although the total length of hospital stay for the index admission was substantially shorter for patients in the on-demand group (mean, 38 days, versus mean, 45 days in the planned group), length of stay on the ward was comparable (mean, 26 and 27 days, respectively; *P *= 0.21).

The on-demand group used substantially less medication and material (for example, days with enterostomy care, blood transfusions). Mean costs per patient for medication were €474 versus €619; for blood products, €89 versus €178; and for enterostomy care, €741 and €917, comparing the on-demand group and the planned-relaparotomy group. Direct medical costs during admission were significantly lower in the on-demand group, with a mean difference of €14,418 (95% CI, €5,274 to €22,983).

Costs associated with readmissions during the 1-year follow-up were comparable (mean number of hospital days, 9.5 versus 11.8 days (*P *= 0.88), with mean costs €4,280 for the on-demand patients versus €5,083 for the planned patients). On average, enterostomy care and associated costs during follow-up were considerable. Patients had an enterostomy for a mean of 140 days (€4,449) in the on-demand group and 150 days (€4,767) in the planned-relaparotomy group (*P *= 0.44).

Distinct differences in resource utilization and associated costs of outpatient and secondary healthcare providers were found for home care supplied by district nurses and stay in rehabilitation centers, in which the on-demand group received less home care (45 hours per patient versus 71 hours in the planned-relaparotomy group; *P *= 0.16) and had shorter stays in rehabilitation centers than did the planned-relaparotomy group (19 versus 23 days; *P *= 0.90). Differences between the study groups in utilization and associated costs of outpatient care and visits to secondary healthcare providers (general practitioner, company doctor) were marginal. The direct medical costs during follow-up (between discharge and 12 months after the index laparotomy) were lower in the on-demand group, with a mean difference per patient of €4,069 (95% CI, €2,660 to €7,063).

Among patients reporting to have paid work, only the occasional patient reported a return to work within 12 months. For all nonresponding patients younger than 65 years, we therefore assumed that they were at least absent for the full friction period (154 days). The mean number of days absent from paid work was estimated as 70 days in the on-demand group versus 50 days in the planned group (*P *= 0.038). Costs associated with lost productivity for these days were estimated as €2,854 and €2,048, respectively.

Overall, for the 1-year study period, mean total costs per patient associated with the on-demand strategy were €65,768 versus €83,450 with the planned strategy (absolute difference, €17,682; 95% CI, €5,062 to €29,004; Wilcoxon-Mann-Whitney test, *P *< 0.005; relative difference, 21%). Of these total costs, 75% were generated during the initial admission, of which 45% to 55% were ICU costs.

Subsequent sensitivity analyses showed that the results of the cost analysis were robust for changes in various assumptions (Table 3). Absolute estimates of total costs were found to change within a limited range for each strategy (<€8,000 for on-demand, and <€10,000 for planned relaparotomy), whereas the relative difference between the two strategies remained stable (21% to 22%).

**Table 3:** Summary of sensitivity analyses: mean total costs and estimated absolute and relative differences between relaparotomy on demand and planned relaparotomy across alternative assumptions and calculation methods

			Mean	Mean		%
	Description	OD	PR	difference	95% CI^a^	Difference
**Analysis**						
Main	Main analysis (most probable assumptions)	65,768	83,450	17,682	(5,062 to 29,004)	21.2%
1	Percutaneous drainage procedures (reimbursement fee as opposed to AMC estimate)	65,754	83,428	17,674	(5,057 to 28,975)	21.2%
2	Ward-stay unit costs (weighted average of Academic and General hospitals)^b^	62,938	81,016	18,078	(5,437 to 28,640)	22.3%
3	ICU-day unit costs (AMC top-down calculation instead of guideline)	70,694	90,980	20,286	(5,959 to 32,160)	22.3%
						
4	With ICU-day unit costs estimated for					
	A United Kingdom	63,235	79,688	16,453	(4,788 to 28,439)	20.6%
	B Germany	61,541	77,172	15,631	(4,578 to 28,037)	20.3%
	C France	69,102	88,401	19,299	(5,371 to 29,721)	21.8%
	D Norway	77,225	100,465	23,240	(5,948 to 31,306)	23.1%
	E Austria	63,794	80,518	16,724	(4,851 to 28,560)	20.8%
	F Canada	58,960	73,338	14,378	(4,223 to 27,415)	19.6%
						
5	Exclude all costs of relaparatomy procedures	62,543	77,913	15,370	(3,018 to 25,395)	19.7%

^a^Based on geometric means; ^b^weighted by ratio of Academic and General hospital beds in the Netherlands (1:6). Explanation of different sensitivity analyses: (1) for percutaneous drainage procedure, AMC-unit costs estimates were replaced by reimbursement fees for this procedure. (2) To avoid the encountered cost differences where direct results from the differentiation between academic and nonacademic hospitals, a weighted average unit cost per hospital ward day was used. This average was weighted by the actual ratio of academic and nonacademic hospital beds in the Netherlands [29]. (3) We differentiated between unit costs of relaparotomies with and those without other surgical procedures (such as enterostomy (re)construction, abscess drainage, colon resection) instead of using the same all-in unit costs for all relaparotomies. (4a through f) To enhance generalizability of the results to other countries with publicly financed health care systems, Dutch reference prices for ICU days were replaced by unit costs estimated for the UK, Germany, France, Norway, Austria, and Canada, respectively [10,19-23]. Additionally (5), we compared the total costs of the two strategies when disregarding the costs of relaparotomy procedures during the index admission because these differences were intrinsic to the strategy itself, as the planned strategy involved more procedures than did the on-demand strategy.

**Figure 1:**
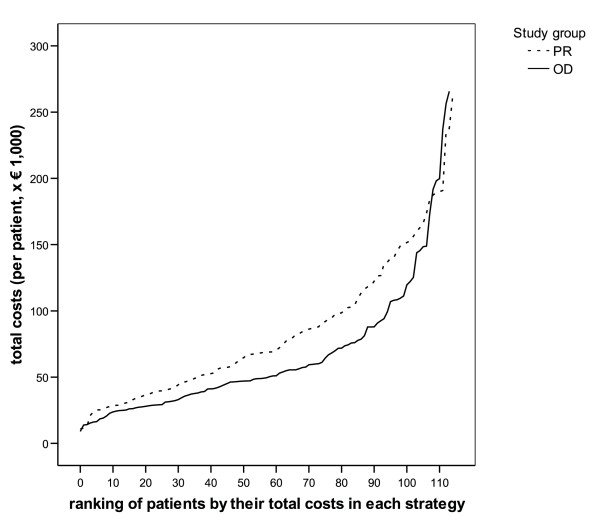
**Comparing on-demand and planned-relaparotomy strategies for patients ranked according to their total costs**. Total costs could be taken as proxy for clinical condition and recovery. The observed difference in total costs per patient was similar for patients with the most favorable conditions and courses of recovery, as compared with patients with more severe conditions or complicated courses of recovery or both. PR, planned relaparotomy; OD, on-demand relaparotomy.

To answer the question whether this difference is consistent across patients with different clinical course, Figure 1 shows the distribution of total costs per patient in each group after patients are ranked according to their total costs. Costs were found to be consistently lower in the on-demand group compared with the planned-relaparotomy group across the whole range of costs, except for a small number of patients at the very high end of total costs.

Relative differences in costs between the on-demand relaparotomy strategy and the planned strategy varied substantially across clinical subgroups: in some subgroups, the mean costs in the planned group are almost twice those in the on-demand group (patients surviving for 12 months versus patients dying within 12 months), whereas costs associated with both strategies appear to be rather comparable in others (for example, anastomotic leakage) (Table 4). In patients who did not survive, 12-months costs were lower in the on-demand group. As none of the formal statistical tests for interaction was significant at the 5% level, the assumption that relative difference in costs between the on-demand and the planned strategy are constant across subgroups has not been rejected.

**Table 4:** Variation in relative differences in total costs between on-demand and planned relaparotomy strategies across various clinical subgroups

	Relaparotomy strategy					
		On demand		Planned	Difference^a^	
	Mean costs p.p.	*n*	Mean costs p.p.	*n*	% from planned	*P *value^b^
Overall	66,216	112	84,152	113	-23.8%	
						
Comorbidity						0.26
No	64,948	48	89,738	41	-32.8%	
Yes	67,168	64	80,971	72	-17.1%	
						
Apache II						0.58
11-20	66,956	96	84,683	94	-22.3%	
>20	61,777	16	81,525	19	-32.3%	
						
Etiology						0.09
Inflammation (1)	40,810	4	81,074	5	-21.7%	
Perforation (2)	62,236	63	88,985	67	-37.0%	
Ischemia (1)	59,591	6	72,080	8	--	
Anastomotic leakage (3)	76,171	35	72,751	27	6.8%	
Other (4)	77,153	4	100,149	6	-6.9%	
						
Elimination of infectious source						0.81
No	71,113	10	90,254	11	-24.2%	
Yes	65,736	102	83,494	102	-18.3%	
						
Localization						0.45
Upper GT (1)	69,828	30	81,146	27	-25.9%	
Lower GT (2)	67,402	70	82,446	74	-42.7%	
Biliary tract (3)	63,496	2	100,115	5	-28.8%	
Appendix (3)	32,075	3	26,575	1	--	
Pancreas (3)	59,352	5	92,446	2	--	
Gynecol (3)	41,650	2	67,720	1	--	
Other (3)			145,821	3	--	
						
Extent of index operation						0.49
1 quadrant	66,079	9	73,381	16	3.0%	
2 quadrants	62,439	34	84,791	26	-30.6%	
Diffuse	68,096	70	86,102	69	-24.8%	
						
Nature of contamination						0.18
Clear (1)	59,306	6	97,791	8	-25.9%	
Turbid (2)	57,179	18	96,672	29	-42.7%	
Purulent (3)	55,746	43	77,003	30	-28.8%	
Fecal (4)	84,707	41	81,977	41	-2.4%	
Bile (3)	40,281	4	61,984	3	-	
						
Survival (at 12 mo)						
No	73,275	80	85,326	72	-17.8%	0.13
Yes	48,569	32	82,089	41	-38.6%	

^a^Model estimates of difference between On demand and Planned relaparotomy strategy based on analyses of geometric means; ^b^For the interaction effect in regression model; (x) numbers between brackets indicate collapsed categories used as interaction effect. The *P *value for the interaction effect in the regression model tests the hypothesis that the relative differences between the two surgical strategies are the same across clinical subgroups (for example, for comorbidity, that -32.8% = -17.1%). *P *values > 0.05 indicate that this hypothesis cannot be rejected at a 5% statistical significance level.

## Discussion

We present an economic evaluation within a randomized clinical trial comparing two commonly used surgical strategies for patients with secondary peritonitis after their initial emergency laparotomy, on-demand relaparotomy and planned relaparotomy. In an earlier publication focusing on the clinical outcomes of the RELAP trial, we demonstrated that patients in the on-demand group did not have a significantly lower rate of poor outcomes compared with the planned group [6]. The results of the detailed cost analyses presented here indicate that, across the full range of healthcare resources, as well as across patients with different disease and recovery courses, resource utilization and associated costs generated by treatment and follow-up of severe abdominal sepsis were substantially lower for the on-demand strategy than for the planned strategy. Furthermore, these relative differences in costs between the two strategies appeared to be quite consistent across a range of clinical subgroups, although for some (for example, anastomotic leakage), this study may have had insufficient power to statistically demonstrate such differences.

The observed cost differences were predominantly related to lengthier ICU stays and duration of mechanical ventilation during the index admission period. Costs of rehabilitation centers and home care and of readmissions to a general hospital during follow-up were also substantial contributors to these cost differences. Although the planned strategy per definition involved at least one relaparotomy procedure, costs generated only by this extra procedure were only a mere fraction of the encountered cost differences. When costs associated with relaparotomy procedures were disregarded, major cost differences between the surgical strategies remained present.

An important component of the total direct medical costs was the ICU stay (often involving mechanical ventilation). Consequently, total costs were highly influenced by the unit costs estimate for an ICU day. We used a reference price based on data from a range of general and academic hospitals in the Netherlands [14]. In the literature, considerable variation was encountered in cost estimates for an ICU stay, if reported at all. This variation due to differences in calculation methods, patient groups, but also in local organization and facilities (staff allocation and remuneration, equipment costs, nonclinical support services and premises) [10] and (national) healthcare system. To enhance the generalizing of our findings to other countries, we presented the consequences of using cost estimates found for the United Kingdom [10], Austria [19], France [20], Canada [21], Germany [22], and Norway [23]. Estimates for countries with publicly funded healthcare systems were better reported in the literature than estimates for countries with other types of healthcare systems (for example, the United States). Information pertaining to these costs and studies addressing the real costs of health care resources appeared to be lacking for non-publicly funded healthcare systems.

In general, resource utilization was found to be higher in the planned group than in the on-demand group. Therefore, adjustments in unit costs would result in changing total costs, rather than affecting the difference between on-demand and planned relaparotomy. Total costs varied to some degree with the different assumptions regarding unit-cost prices, but the relative difference between the strategies remained consistent across these analyses. On average, the on-demand strategy generated approximately 21% less costs than planned relaparotomy. Per 1,000 patients admitted to an emergency room with severe peritonitis, half of whom are currently operated on according to the planned strategy, some €10 million could be saved.

No other studies have reported a detailed description of costs associated with resource utilization generated by abdominal sepsis patients treated by either strategy, and we can compare volumes only; our findings were comparable to those of a retrospective study comparing on-demand and planned relaparotomy, reporting similar figures for average length of hospital stay (49.5 vs. 52.0 (including ICU) and average length of ICU stay (12.6 versus 17.8 days). Duration of mechanical ventilation was a few days longer (10.3 versus 13.9) as compared with our results (8.5 versus 12 days), but the difference (on-demand, 2.5 days shorter ventilation period) was consistent with our findings [3,6].

This study has some strengths and limitations. First, the economic evaluation was performed as part of a randomized, controlled trial that stratified for severity of disease, ensuring that the patients in both strategies were comparable with respect to clinical and prognostic factors. Differences in resource utilization and related costs can therefore confidently be attributed to the surgical strategy. Furthermore, the economic evaluation was based on data on resource utilization required for the clinical trial and extended with additional relevant information acquired with self-administered questionnaires. This bottom-up strategy provided insight into the healthcare process and main cost-driving factors. Although the majority of costs were generated during index admission, systematic documentation during follow-up demonstrated that these differences remain unchanged from a societal perspective.

Currently, support of the on-demand strategy is growing [24-27], and sound empiric evidence regarding the optimal approach is now available from a prospective randomized comparison. The clinical results of the RELAP trial, reported elsewhere [6], and the present economic evaluation support further implementation of an on-demand relaparotomy strategy for treatment of patients with abdominal sepsis.

## Conclusions

This economic evaluation prospectively demonstrated that resource utilization and associated costs generated during treatment and follow-up of severe peritonitis were substantially lower for an on-demand strategy compared with a planned strategy. These differences were found across the full range of healthcare resources as well as across patients with different courses of disease. Considering that patients in the on-demand relaparotomy strategy group had a lower (albeit not statistically significant) rate of adverse outcomes compared with the planned-relaparotomy group [6], the reduction in costs (21%) associated with healthcare utilization renders the on-demand relaparotomy a more efficient surgical strategy in patients with severe peritonitis. Implementation of an on-demand relaparotomy strategy could have a positive impact on the healthcare expenses for this severe and costly medical condition.

## Key messages

• Patients with severe peritonitis require intensive monitoring and medical treatment, often including lengthy ICU stays, and therefore incur substantial costs to the healthcare system.

• The first randomized clinical trial comparing on-demand versus a planned-relaparotomy strategy in patients with severe peritonitis (RELAP trial) indicated no clear differences in primary clinical outcomes.

• To assess the economic impact of differences in resource use, we performed a full economic evaluation from a societal perspective alongside this trial.

• Mean total costs per patient were 20% lower in the on-demand group as compared with the planned group.

• The substantial difference in costs renders the on-demand strategy a far more efficient relaparotomy strategy in patients with severe peritonitis.

## Abbreviations

APACHE: Acute Physiology and Chronic Health Evaluation; CRF: clinical report form; CEA: cost-effectiveness analysis; CMA: cost-minimization analysis; CT: computed tomography; FFP: fresh frozen plasma; HR-QoL: health-related quality of life; ICU: intensive care unit; OD: on demand relaparotomy; PCD: percutaneous drainage; PR: planned relaparotomy; RELAP trial: randomized controlled trial comparing relaparotomy on demand with planned relaparotomy; US: ultrasound; US$: United States dollar; 95% CI: 95% confidence interval.

## Competing interests

The authors declare that they have no competing interests.

## Authors' contributions

MAB, DJG, HO, JBR, and CAJMB designed the clinical study. BCO, CAJMB, and MAB designed the economic evaluation alongside the clinical trial. OR and CWM were responsible for the coordination of the study, including contacting patients and collecting and entering data. KRB and OR were responsible for assessment and processing of follow-up data. BCO, KRB, OR, JBR, and MAB were responsible for the cost analyses. HGG, PWG, BL, MFG, and EPS were responsible for including more than 10% of the randomized patients in their participating hospital. BCO analyzed data and prepared initial versions of the manuscript. BCO, KRB, OR, JBR, and MAB were responsible for the final manuscript. BCO, KRB, OR, JBR, and MAB interpreted and discussed all data. All authors read, reviewed, and approved the final manuscript.

## Supplementary Material

Additional file 1 **Table reporting units of resource use, unit costs (€), valuation method and volume source used for the cost analyses**. Click here for file
