# Supplementary material for: Costs of relaparotomy on-demand versus planned relaparotomy in patients with severe peritonitis: an economic evaluation within a randomized controlled trial
Source: Crit Care. 2010 May 27;14(3):R97. doi: 10.1186/cc9032 (PMC2911734; doi:10.1186/cc9032)
Supplement: Additional file 1 — Table reporting units of resource use, unit costs (€), valuation method and volume source used for the cost analyses. [file cc9032-S1.DOC]

| **Cost category** | **Unit** | **Unit cost** | | | | | | **Valuation** | | **Volume source** | | |
| --- | --- | --- | --- | --- | --- | --- | --- | --- | --- | --- | --- | --- |
|  | **method (source)** | | | | | | | | | | | |
| **direct medical** |  | | | | | | | | | | | |
| **Admission** |  | | | | | | | | | | | |
| hospital stay - General hospital | day | | 344 | | | | | | guideline (1) | | | CRF |
| hospital stay - Academic hospital | day | | 486 | | | | | | guideline (1) | | | CRF |
| ICU-stay - General hospital | day | | 1719 | | | | | | guideline (1) | | | CRF |
| ICU-stay - Academic hospital | day | | 2128 | | | | | | real costs (2) | | | CRF |
|  |  | | | | | | | | | | | |
| **Surgical procedures** |  | | | | | | | | | | | |
| laparotomy (index procedure) | procedure | | | | | 2434 | | | real costs (3) | | | CRF |
| relaparotomy | procedure | | | | | 2434 | | | real costs (3) | | | CRF |
| elective relaparotomy | procedure | | | | | 2434 | | | real costs (3) | | | CRF |
| percutaneous drainage | procedure | | | | | 298 | | | real costs (3) | | | CRF |
| enterostomy | patient | | | | | 2338 | | | real costs (2) | | | CRF |
|  |  | | | | | | | | | | | |
| **Diagnostic procedures and cultures** | | | | | | | | | | | | |
| CT | procedure | | | | | | 250 | | real costs (3) | | | CRF |
| US | procedure | | | | | | 87 | | real costs (3) | | | CRF |
| X-abdomen | procedure | | | | | | 44 | | real costs (3) | | | CRF |
| microbiology | culture | | | | | | 14 | | tariff (4) | | | CRF |
|  |  | | | | | | | | | | | |
| **Medication and other materials** |  | | | | | | | | | | | |
| antibiotic therapy | day | | | * | | | | | real costs (5) | | | CRF |
| mechanical ventilation | day | | | 369 | | | | | tariff (4) | | | CRF |
| enterostomy care | day | | | 31 | | | | | real costs (2) | | | CRF |
| blood products |  | | | | | | | | | | | |
| packed cells | unit | | | 183 | | | | | guideline (1) | | CRF | |
| FFP | unit | | | 157 | | | | | guideline (1) | | CRF | |
| platelet transfusions | unit | | | 83 | | | | | guideline (1) | | CRF | |
|  |  | | | | | | | | | | | |
| **Other health care providers** |  | | | | | | | | | | | |
| outpatient visit - General hospital | visit | | | | 57 | | | | guideline (1) | | questionnaire | |
| outpatient visit - Academic hospital | visit | | | | 102 | | | | guideline (1) | | questionnaire | |
| general practitioner | visit | | | | 21 | | | | guideline (1) | | questionnaire | |
| company doctor | visit | | | | 23 | | | | ** | | questionnaire | |
| paramedical | visit | | | | 23 | | | | guideline (1) | | questionnaire | |
| district nurse | hour | | | | 41 | | | | guideline (1) | | questionnaire | |
| rehabilitation facility | day | | | | 343 | | | | guideline (1) | | questionnaire | |
|  |  | | | | | | | | | | | |
| **direct non-medical** |  | | | | | | | | | | | |
| travel costs | km | | 0.18 | | | | | | guideline (1) | | CRF/questionnaire | |
|  |  | | | | | | | | | | | |
| **indirect** |  | | | | | | | | | | | |
| absence from paid work | day | | | *** | | | | | guideline (1) | | questionnaire | |
